# PReS-FINAL-2020: Cell type specific transcriptome analysis in patients with enthesitis related arthritis category of juvenile idiopathic arthritis (JIA-ERA)

**DOI:** 10.1186/1546-0096-11-S2-P33

**Published:** 2013-12-05

**Authors:** A Aggarwal, A Myles, P Gaur

**Affiliations:** 1 Clinical Immunology, Sanjay Gandhi Postgraduate Institute of Medical sciences, Lucknow, India

## Introduction

Enthesitis Related Arthritis Category of Juvenile Idiopathic Arthritis (JIA-ERA) is the most common category of JIA seen in Asian Indians. Transcriptome analysis is a useful tool to analyse pathways involved in disease pathogenesis. Peripheral blood mononuclear cells (PBMC) and SFMC analysis showed involvement of innate immune cells in JIA-ERA. However PBMC/SFMC have variable number of different cells and that can affect interpretation. No data is available on cell type specific transcriptome analysis of blood and synovial fluid in children with JIA-ERA.

## Objectives

To study the cell type specific transcriptome analysis of blood and synovial fluid in children with JIA-ERA.

## Methods

Six samples each of peripheral blood and synovial fluid were collected from patients with ERA-JIA. Blood from 6 healthy controls was also collected. Mononuclear cells were separated by density gradient centrifugation. B cells, T cells and monocytes were separated using MACS columns and purity assessed by flow cytometry. After RNA extraction and checking the quality of RNA (RIN > 8) microarray was done using Illumina chips WG 12 for whole PBMC/SFMC population, T cells, B cells and monocytes. Some of the significant genes were validated by qRT-PCR.

## Results

Unsupervised hierarchical clustering revealed that cell subsets could be distinguished based on their gene expression profile. No significant differences were observed between PBMC of patients and healthy controls. Comparison of SFMC and PBMC reconfirmed the results seen earlier. Among T cells and B cells the differential athways identified were related to inflammation like Cell adhesion, antigen processing, cytokine and chemokine signaling,BCR signaling and leukocyte migration.Results obtained with monocytes are summarized below in table 1.

**Table 1:** 

Groups compared	Genes up regulated	Genes down regulated	Number of dysregulated pathways [total (significant)]	Pathways of immunological relevance
EB vs EF	776	189	19 (12)	Cell adhesion, IgA production, antigen processing, lysosomal processing

CBMO vs EBMO	821	1251	21 (12)	Cytokine signaling, TLR signaling, antigen presentation, chemokines signaling

EBMO vs EFMO	595	512	17 (9)	Complement cascade, cytokine signaling, antigen presentation

EB: ERA blood mononuclear cells, CB: control blood mononuclear cells, EF: ERA Synovial fluid mononuclear cells, Mo: CD14+ monocytes, The major differences were found in monocyte subset. TLR pathway was one of the major pathway identified besides antigen presentation, cytokine and chemokine signalling.

## Conclusion

Monocyte probably play a major role in pathogenesis of JIA-ERA and TLR signalling may be the pathway involved.

## Disclosure of interest

None declared.

